# Scoping review of the societal impacts of compound climate events

**DOI:** 10.1007/s44274-025-00185-y

**Published:** 2025-01-16

**Authors:** Caroline A. Fehlman, Sophia C. Ryan, Kristen G. Lysne, Quinn M. Rundgren, Taylin J. Spurlock, Rustyn O. Orbison, Jennifer D. Runkle, Margaret M. Sugg

**Affiliations:** 1 Department of Geography and Planning, Appalachian State University, Boone, NC USA; 2 Department of Mathematical Sciences, Appalachian State University, Boone, NC USA; 3 North Carolina Institute for Climate Studies, North Carolina State University, Asheville, NC USA; 4 Rankin Science West, Appalachian State University, Boone, NC 28607 USA

**Keywords:** Compound climate events, Recurrent compound climate events, Public health, Agriculture, Land-use, Built environment

## Abstract

Climatic extremes have historically been seen as univariate; however, recent international reports have highlighted the potential for an increase in compound climate events (e.g., hot and dry events, recurrent flooding). Despite the projected increase in the frequency of compound climate events and the adoption of compound event terminology, few studies identify climate extremes as compound climate events and little evidence exists on the societal impacts of these compound climate events. This scoping review summarizes key findings and knowledge gaps in the current state of empirical studies that focus on the societal impacts of compound climate events. We identified 28 eligible studies published in four databases reporting on the societal impacts of compound climate events in four sectors: agriculture, public health, the built environment, and land use. Overall, we found the need for more research explicitly linking compound climate events to societal impacts, particularly across multiple compound climate events, rather than single case study events. We also noted several key findings, including changes in agricultural productivity, loss of habitat, increased fire risk, poor mental health outcomes, decreased health care access, and destruction of homes and infrastructure from these events. Additional research is needed both globally and locally to understand the implications of compound climate events across different geographic regions and populations to ensure responsive adaptation policies in a compound climate event framework.

**Supplementary Information:**

The online version contains supplementary material available at 10.1007/s44274-025-00185-y.

## Introduction

Current and future impacts from climate change are projected to increase the earth’s temperature by 1.5C or more by the end of the century, resulting in highly likely changes in the frequency and intensity of flooding, heatwaves, droughts, wildfires, and severe weather events [1]. These increases in extreme climate events are impacting food security, terrestrial and coastal ecosystems, human health, agriculture, and infrastructure within overburdened and frontline communities (e.g., low-income, women and children, rural areas) experiencing the brunt of the impacts [1]. Climate extremes have historically been investigated as univariate and singular events, and the conventional approach is to examine each climate extreme in isolation [2]. However, scientists increasingly acknowledge overlapping and co-occurring physical climate events and the increasing potential for compound climate events (CCEs) [3]. Most recently, CCEs such as the 2024 Hurricane Helene and the severe flooding in Western North Carolina have had significant impacts [4]. Additionally, recurrent CCEs like Hurricanes Helene and Milton in Florida have led to widespread power outages, community devastation, and over 200 fatalities [4, 5].Other examples include 2024 Hurricane Beryl and extreme heatwaves in Texas, which resulted in a loss of electricity and 36 deaths [6]; Hurricane Ida in 2021, hitting communities already impacted by Hurricanes in 2020 [7, 8]; and Hurricanes Irma and Maria in 2017, which impacted maternal health outcomes in Puerto Rico and the U.S. Virgin Islands [9–11]. Compound flooding also occurred in the Northeastern US in 2021 (55 deaths, $21.4 billion dollars in damage) and heat, drought, and wildfires in 2021 in the Western US ($38 billion dollars in damage) [12]. These compound events are expected to become more frequent with climate change [13].

By examining CCEs, we seek to understand how future research can address the societal implications of these events, which are projected to increase in frequency [13, 14]. More specifically, we aim to identify empirical research on the implications of CCEs for the sectors of agriculture, public health, the built environment, and land use. We employ Arksey and O’Malley’s [15] methodological framework for scoping review to identify relevant studies, chart the data, and collect, summarize, and report results. We understand our limited definition limits our findings. Yet, our work provides one of the first attempts to identify existing literature that directly studies CCEs and the societal implications of extreme climate events that have the potential to co-occur, either spatially or temporally. Our results highlight the complexities and nuances of dealing with CCEs, which we hypothesize result in more adverse societal outcomes; in addition to providing valuable insights for future research and a more holistic understanding of the impacts of these events.

### Definitions of compound climate events

Compound events, or multiple climatic events of similar or different types, were first defined in a special report from the Intergovernmental Panel on Climate Change (IPCC) [16] as multiple extreme or non-extreme events occurring (1) simultaneously at the same place; (2) concurrently across multiple regions; or (3) in rapid sequence, in the same location, and are now a focal point of emerging research. Despite a formal IPCC definition, the scientific community has been slow to adopt this definition, and previous studies have highlighted the abundance of terms to quantify climate event interrelation [17–19]. The complexity of many interrelated extreme weather events makes it difficult to develop a widely applicable compound event attribution framework, and methodologies to assess the societal impacts of these CCEs remain largely unknown. This lack of a common methodology is due to the diverse nature of CCEs, the soft boundaries of events (not all events fit perfectly into the presented categories, and some cannot be easily assigned to a single type, necessitating soft boundaries), and deep knowledge required of the local climate system through the understanding of the causal structure of compound events (e.g., specific drivers, modulators) and their underlying phenomena [20, 21].

In addition, compound events have been considered within the context of disasters, or extreme impacts suffered from hazardous exposures, which interact with underlying resilience and adaptation at the local level to alter the normal functioning of a community [22]. These disasters include non-climate events such as the COVID-19 pandemic [23] and earthquakes [24]. Emerging research at the intersection of climate and society has begun to link the impacts of multiple disaster exposures (e.g., hurricanes, earthquakes, terrorist attack, oil spills), showing direct and indirect effects on health of the community [25]. Other reviews that have focused on “multi-hazard risk assessment” demonstrate the majority of studies focus on landslides, with an underrepresentation of meteorological and hydrological events (i.e., heatwaves, coastal/riverine flooding, and tropical cyclones), and therefore recommends future research that examines “the cascading and intertwined relationships of these events” [26].

Zschesicler et al. are among the first to lay out the typology, or classification, of CCEs, addressing the characteristics (e.g., hazards, drivers, and modulators), categories (e.g., preconditioned, multivariate, temporal compound, spatial compound), and methodologies and assessments for measuring CCEs and their impacts [21]. Furthermore, Zschesicler recommends bottom-up approaches that focus on specific events and case studies to improve understanding of the drivers of compound events and associated risks [20, 21]. These localized approaches are especially needed for linking sectoral impacts to physical hazards [21]. By adopting a localized approach and examining the interactions among multiple CCEs and the interplay of various factors like exposure and underlying vulnerability, scientists can more accurately evaluate risks. This method of risk assessment that includes CCEs thereby addresses the common underestimation or overestimation of risks that occur when only single climatic events are considered [24, 27, 28].

At the time of this article, climatic studies have typically focused on the observed spatial and temporal frequency of CCEs [12, 14, 29] and, in some cases, the future projections of these events [30, 31]. Few studies have explicitly examined the societal implications of CCEs. Our work builds upon one of the few scoping reviews to address this research gap, [25], which focuses on empirical research on the public health implications of multiple disasters, rather than CCEs, and define these events as either (1) “cascading disasters (disasters generating secondary disasters),” (2) “compound disasters (combinations of simultaneous or successive extreme hazard events)” and (3) “recurrent disasters (in which the same hazard repeats).” Unlike Leppold et al., we focus more broadly on the societal implications of multi-climatic events or CCEs by excluding non-climate events, including man-made (i.e., 9/11 terrorist attacks), technological (i.e., industrial accidents), and geologic events (i.e., earthquakes) and focus only on empirical studies directly related to the impacts of CCEs [25]. We define CCEs using the US National Climate Assessment (2023), which defines CCEs as climate extremes that occur simultaneously or consecutively and that exacerbate the societal and ecosystem impacts that typically occur from individual climate hazards [13]. Furthermore, we define CCEs as extreme climate events occurring simultaneously (spatial) or consecutively (temporal and/or recurrent), similar to Zscheischler et al. 's [21] typology of compound events. Our definitions are depicted in Table 1, and include a visual representation and example of each CCE type. We emphasize climate hazards (e.g., droughts, heatwaves, and wildfires) as a characteristic of compound events. Like Leppold et al., we consider recurrent climate events as a type of temporally compound event that includes the same climate event (e.g., flood) [25, 32, 33]. Other definitions, including cascading risks (e.g., extreme events, in which cascading effects increase in progression over time and generate unexpected secondary events of strong impact) [34], are also found in the literature [25], particularly in research around the built environment [35], but like Singh et al. [12] and Zscheischler et al. [21], these events were excluded from our review to ensure the focus on CCEs.

**Table 1:** Definitions for compound climatic events (CCEs) that were adopted in this paper [12, 16, 19, 21, 25]

Name	Adopted cross-disciplinary definition	Established definition(s)	Examples	Illustrative example
Temporal compound	Multiple extreme climate events that occur in the exact same location at different time points, meaning that the climate hazards are temporally mutually exclusive	“In rapid sequence, in the same location” [16]; “successive hazards in a location” [12]; “succession of hazards that affect a given geographical region, leading to, or amplifying, an impact when compared with a single hazard” [21]	A heatwave is followed by a flood or tropical cyclone	
Recurrent	Repeat exposures to the same climate disaster; considered a sub- type of temporal compound disaster	“The recurrence of a single natural hazard in the same geographic region over a one-year period” [25, 32, 33]	A hurricane hits the same city twice within a year	
Spatial compound	Multiple extreme climate events occur in the same area and approximately the same time frame, meaning that the events are overlapping and interacting in space–time	“Simultaneously at the same place.” [16]; “similar or disparate hazards occurring simultaneously or within a short time window” [12]; “when multiple connected locations are affected by the same or different hazards within a limited time window,” [21]	Drought and Heatwave events at the same time	

## Methods

From January through February 2023, a systematic search was conducted across ScienceDirect, PubMed, Google Scholar, and EBSCO electronic databases to identify relevant peer-reviewed publications, following PRISMA guidelines for scoping reviews [15, 36]. A search string was adapted for each database through several iterations of preliminary searches and co-author discussions among authors (Supplemental Table 1). Search strategy and selection criteria aimed to map the societal impacts of CCEs across one or more of the following domains outlined in the Lancet Countdown for Health and Climate Change: CCE impacts, exposures, and burdens; adaptation, planning, and resilience for health; mitigation actions and health co-benefits; financial and economic impacts; and public and political engagement [37, 38]. Inclusion criteria included: empirical original research; the publication year must be from 2001 onwards for relevance to societal impacts in recent decades; and in the English language. In addition, all included articles had to focus on CCEs as their main objective, with analysis including empirical research on the direct societal implications of societal CCEs. Studies that focused on projections and predictions of CCEs were excluded. Papers were sorted into categories based on societal impacts for the following sectors: agriculture, public health, built environment, and land use change. These categories were used to guide search terms and inclusion and exclusion criteria.

**Fig. 1:**
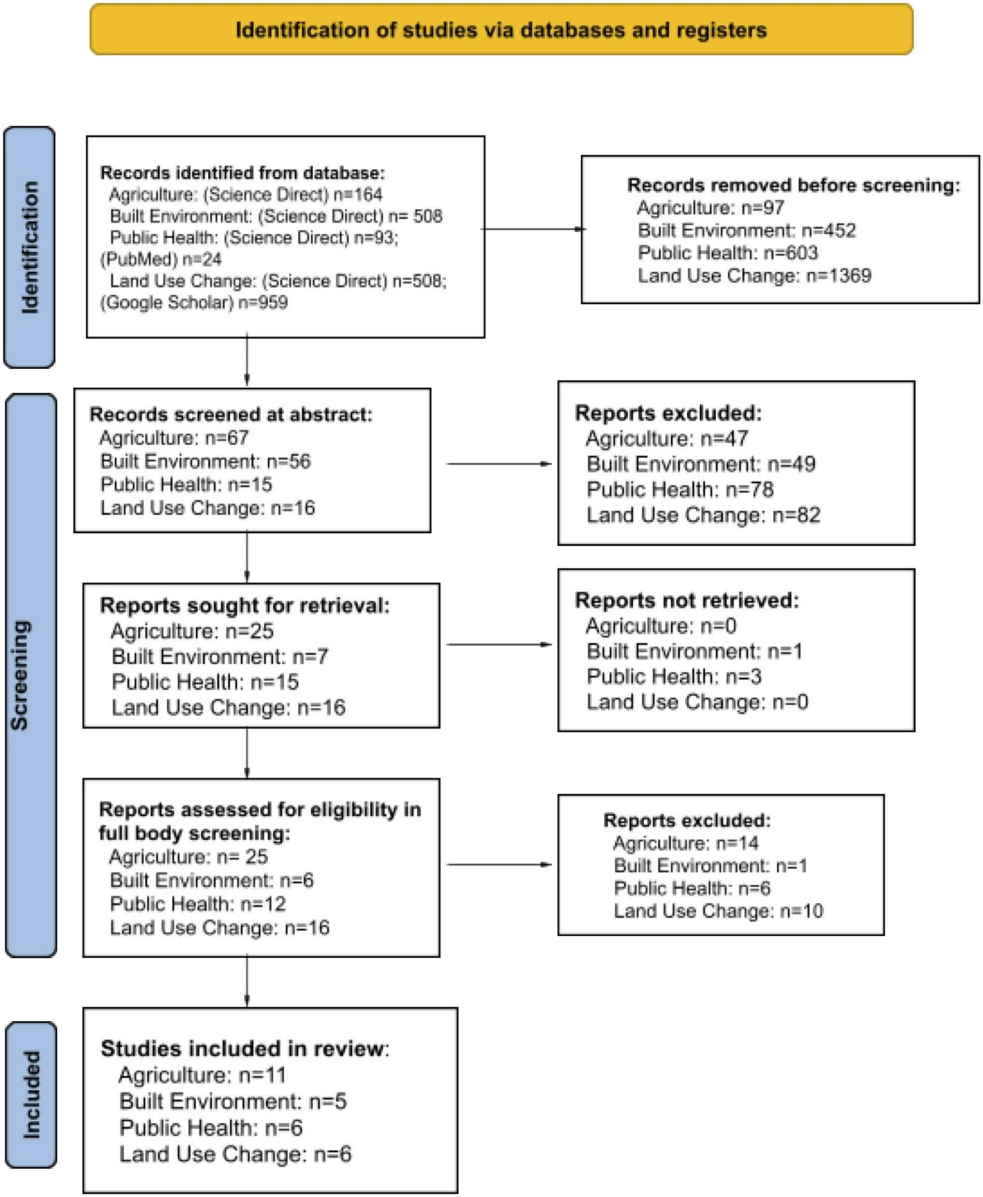
The Preferred Reporting Items for Systematic reviews and Meta-Analyses extension for Scoping Reviews (PRISMA-ScR) [36] were used to track the papers obtained, reported, screened, and excluded in our scoping review using our inclusion criteria

Data abstraction consisted of two authors conducting title/abstract and full-text screening and two authors independently extracting data from studies that met the inclusion criteria into a Microsoft Excel sheet, for each societal sector. Discrepancies were addressed in weekly meetings for all co-authors through discussion to ensure agreement about article inclusion and data extraction. Articles that did not meet the strict inclusion criteria were removed from the sample based on abstract screenings and co-author discussions (Fig. 1).

## Results

**Fig. 2:**
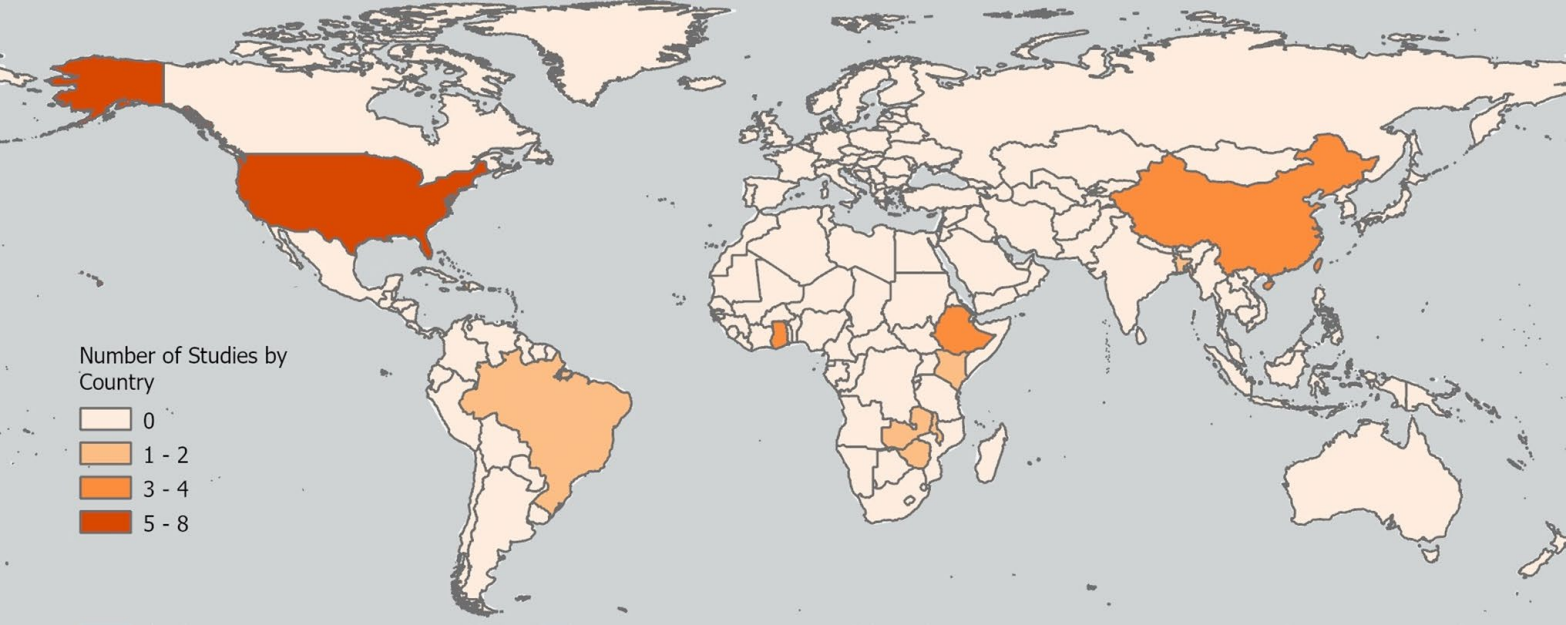
Spatial distribution of studies included in this scoping review. Studies that did not specify a country focal area (n = 3) [39, 63, 66] were excluded from the map. The Mendez-Lazaro et al. [54] study was focused on Puerto Rico and the U.S. Virgin Islands and was therefore included with the count for the United States

We included 28 studies in this scoping review that were published between 2001 and 2023 (Fig. 1). Overall, we found few studies that explicitly study CCEs in the context of societal impacts. Most of the studies were conducted in the United States (n = 8) and China (n = 4) (Fig. 2). The majority of the studies used a mixed-methods approach (n = 16), though some reported purely quantitative methods (n = 7) or qualitative methods (n = 5). Of the societal impacts, agriculture had the most studies included in this scoping review (n = 11).

Recurrent CCEs (n = 15), specifically recurrent flood events (n = 7) and drought events (n = 6), are highlighted as the most discussed CCE exposure. Within those papers, agriculture (n = 8) dominated the focus on recurrent events, followed by the built environment (n = 3). Five studies were temporal CCEs, and the remainder (n = 8) were spatial CCEs. Spatial CCEs dominated studies in land-use change (n = 4). Studies related to public health included recurrent CCEs (n = 3), spatial (n = 2) and temporal CCEs (n = 1).

**Fig. 3:**
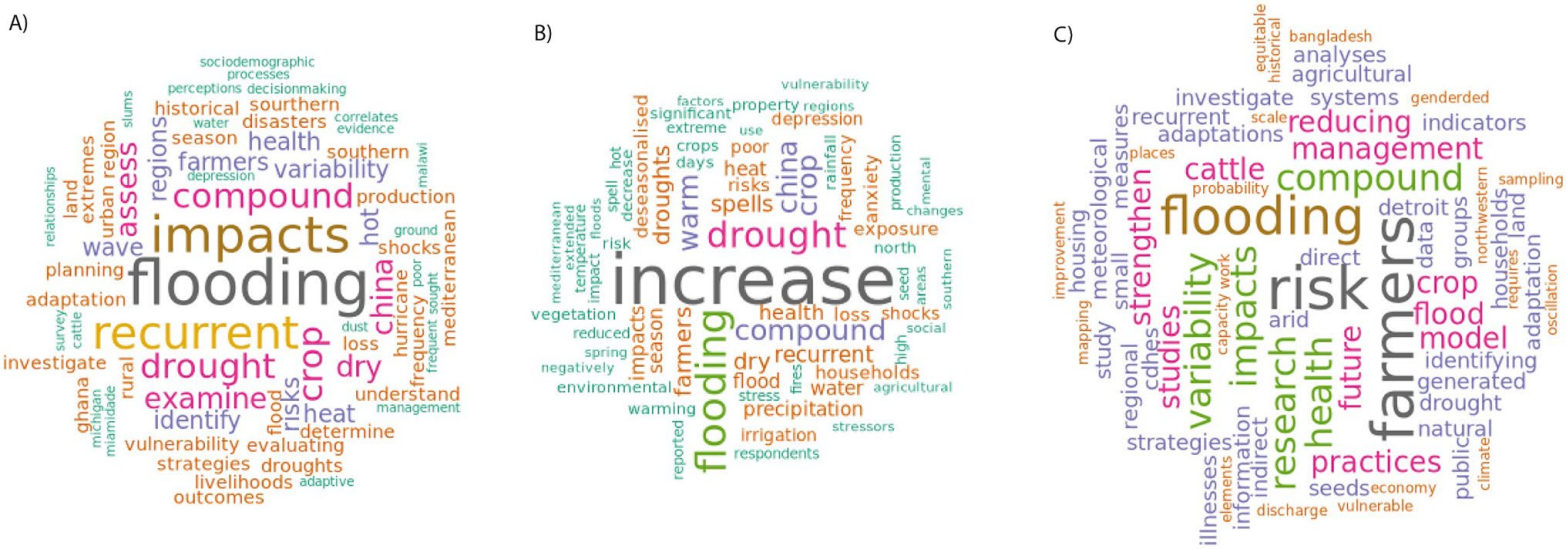
Word cloud of the most used words in the studies included for **A** Objectives, **B** Findings, and **C** Future Research. Word phrases were extracted from the Excel data chart, and font size and shading represent the most frequent words found in articles for each column of the data chart (objectives, findings, and future research)

Flooding (word occurrence = 20), drought (word occurrence = 21), and CCEs (word occurrence = 15) were highlighted as the major topics of discussion throughout the papers (Fig. 3). Studies also emphasized impacts (n = 6), recurrent events (n = 6), and crops (n = 5) as a major focus of the study.

### Agriculture

From the included studies for agriculture (n = 11), a majority (n = 8) examined the effects of recurrent drought on agricultural systems and livelihoods [39–46], while few addressed exposure to recurrent floods, heat waves, or compound temporal CCEs of heat waves and droughts [47–49]. The largest share of the research initially downloaded and viewed during the scoping process (n ~ 75) either centered on agricultural systems or focused on food supply with compound socio-economic events (e.g., political conflicts, COVID-19, food supply chain) and were thus excluded from this review.

A large portion of the studies (n = 7) employed mixed-method research designs [40, 41, 43–46, 48]. For instance, researchers utilized random sampling to gather primary data through household and farmholder surveys and semi-structured interviews. Secondary data sources included meteorological, soil, and socio-economic data. The majority of studies (n = 8) employed longitudinal study designs [41–48] and three studies were cross-sectional [39, 40, 49].

The findings consistently demonstrated that recurrent drought CCEs are becoming more frequent and intense, leading to adverse effects on agricultural systems [39, 40, 42, 44] and on the livelihoods of farmholders [41, 43, 45]. Regions experiencing recurrent drought have reported declines in cattle populations [41, 43], increases in food insecurity [45], and long-lasting stress on crop yield [41].

#### Crops and livestock

Six of the included papers identified recurrent drought as the most prevalent CCEs impacting crop yield and productivity [39, 40, 44–46, 49], while two [41, 43] investigated recurrent drought impacts on cattle. Lei et al. [46] and Derbile et al. [44] both found recurrent droughts had the most frequent negative impacts on crop production, notably rice production in China [46] and maize and rice production in Ghana [44] due to disruption of irrigation supply. Similarly, recurrent drought emerged as a significant CCE affecting cattle production [41, 43]. Recurrent drought was significantly associated with a decline in cattle production and population in Ethiopia and Kenya, with the authors highlighting lasting recovery efforts as amplifying the negative impacts of recurrent drought exposure [41, 43].

#### Food security

Few studies have examined the impact of CCEs on food security, with the exception of Hilemelekot et al. [45], who investigated the effects of recurrent drought on household food security in rural Ethiopia (1981–2016). Food insecurity was linked not only to recurrent drought but limited access to adaptation measures such as soil and water conservation, agroforestry, livelihood diversification, and drought-resistant crops. Socio-economic factors, including advisory services, training access, farm size, gender, and credit availability, were also identified as key determinants [45].

#### Perceptions

Chidanti-Malunga [41] and Mubaya et al. [48] examined farmer perceptions of climate impacts on their livelihoods, farming practices, and the adaptations that have enhanced their resilience to recurrent seasonal flooding and prolonged droughts. In Zambia, most farmers in the study (70%) were aware of changes in climate patterns, and 72–85% of farmers reported increased experiences of recurrent flooding and heavy precipitation events CCEs [42]. Farmers in Southern Zambia and Southwestern Zimbabwe highlighted the influence of socio-economic and spiritual factors on farmers' perceptions of climate variability, emphasizing the significance of environmental management in the farming community [48].

### Public health

Six studies were included; the majority were longitudinal (n = 5), and one was cross-sectional. Three of the studies investigated recurrent CCEs [50–52], while two investigated compound spatial events [53, 54], and the remaining one investigated a compound temporal event [55]. Identified studies considered the impacts of compound drought and flood events [55], hurricanes [50, 54], recurrent flooding [51, 52], and compound heat and flooding [53] on public health.

#### Chronic and infectious disease health

The three studies that investigated impacts on chronic and infectious disease found exposure to compound flooding and drought [55], repeated hurricane exposures [54], and exposure to recurrent flooding [51] were significantly associated with negative impacts to general health. Health outcomes for CCEs were diverse and included asthma [51] and infectious disease [54, 55]. In particular, recurrent household flooding in Detroit, Michigan, was significantly associated with pediatric and adult asthma prevalence; recurrent flooding was most common for renters and in homes with poor infrastructure (i.e., leaking ceilings, basement cracks) [51]. In Ghana, residents in informal settlements exposed to recurrent flooding and drought were significantly linked to an increase in infectious diseases, and recurrent drought was associated with a higher prevalence of heat-related illnesses [55]. Recurrent hurricane exposure was associated with an increase in exposure to poor water quality and vector-borne diseases among gynecological cancer patients in Puerto Rico and the US Virgin Islands [54]. Overall, studies found that health impacts from CCEs disproportionately impact individuals living in poor housing conditions [51, 55] and those with pre-existing health conditions [54].

#### Mental health

Exposure to CCEs was consistently associated with increased poor mental health outcomes [52, 53, 55]. In Ghana, recurrent flooding was significantly associated with anxiety, fear, and chronic stress, particularly in women and children. Underlying social determinants also enhanced vulnerability and were noted as: housing type, class and wealth, family size, and social relations [52]. Exposure to compound flooding and drought in Ghana was also significantly associated with an increased prevalence of depression, specifically as a result of property loss [55]. Research in Bangladesh found that 12 months following exposure to recurrent flooding, individuals were associated with increased odds of co-occurring mental health outcomes, like depression and anxiety [53]. Repeat exposure to recurrent hurricanes in Puerto Rico and the US Virgin Islands was associated with increases in mental distress among gynecological cancer patients [54].

#### Healthcare access as a social determinant of health

Exposure to recurrent hurricanes is significantly associated with interruptions to healthcare access among populations and distress among healthcare providers [50, 54]. Méndez-Lázaro et al. [54] investigated the impacts of Hurricanes Irma and Maria on women with gynecological cancer in Puerto Rico and the US Virgin Islands. Results from this study highlight that women with gynecological cancers were more vulnerable to a disruption of essential services [54]. Herberman Mash et al. [50] investigated how healthcare providers were impacted by repeat hurricane exposure in Florida, USA. The majority of healthcare workers (87%) reported difficulty managing work-life balance during and in the immediate aftermath of a hurricane, and 43% of workers did not receive disaster-specific training. Both studies found that CCEs significantly impacted healthcare access but differed in the populations of interest (i.e., cancer patients vs healthcare workers).

### Built environment

Five studies were included that investigated the effect of CCEs on the built environment. CCEs that cause damage to infrastructure include hurricanes [56] and extreme flooding [57–60]. Two of the studies investigated compound temporal events [56, 57] and three studies looked at recurrent events [58–60]. Three studies were longitudinal [57, 58, 60] and two were cross-sectional [56, 59]; two of the studies used a qualitative design [58, 59] and three studies used a mixed-method design [56, 57, 60].

#### Infrastructure impacts

McGreevy et al. [56] followed farmers in rural Haiti in the aftermath of Hurricane Matthew (2016–2019). The long drought following Hurricane Matthew destroyed structures, crops, and irrigation infrastructure, hindering the community's ability to recover effectively [56]. In Imperial Beach, California, recurrent flooding from 1970 to 2021 has become a high-risk hazard for local residents due to the lack of floodwater infrastructure [57]. The confluence of seawater and groundwater intrusion accompanied by compound flooding could potentially increase flooding in the area by time and depth. The risk of flooding is increasing, such that both one-year and hundred-year rainfall events in Imperial Beach could destroy the entire flood management system [57].

#### Personal property impacts

Recurrent flooding can also reduce property value, particularly in low-lying areas [58]. Sea-level rise and heavy rain events have caused recurrent tidal floods, leading to an estimated loss of $115 million of real estate value since 2005 in Miami-Dade, Florida [58]. The compound effect of sea-level rise and heavy rains, coupled with properties in low-lying areas and poor drainage systems, resulted in widespread flooding. The increasing incidence of flooding impacts the real estate industry by decreasing or restricting property value appreciation [58]. Additionally, cases of recurrent flooding have resulted in compound coastal water events (CCWE) in vulnerable communities in rural eastern North Carolina, as reported by emergency managers, planners, and public officials [59]. The risk of CCWE in rural eastern North Carolina has created a three-pronged problem of pluvial, fluvial, and coastal flooding [59]. The primary reasons for an increased risk in the community due to the recurrent CCWE flooding were deteriorating stormwater infrastructure, failure to adhere to proper land management practices, and a lack of foolproof flood gauges, stormwater models, and updated engineering standards.

#### Flood control impacts

Flooding in Bangladesh was exacerbated following Cyclone Mora in 1991 due to a series of tidal floods accompanied by extreme precipitation events that continued for over 30 years [60]. These recurrent events have put a strain on Bangladesh officials to implement structural integrity measures to minimize the impacts of recurrent flooding. In particular, the most significant threat of flooding has impacted Chittagong City, where it is recommended that a sluice gate should be placed to assist the pumping facility [60].

### Land-use change

Land-use change caused by CCEs increases social vulnerability, loss of habitat, and loss of production. Six studies were included in the data extraction for land use change; the majority of studies were longitudinal (n = 5) [61–65], and one study was cross-sectional [66]. Four studies investigated compound spatial events [61, 62, 64, 66], one study investigated compound temporal events [65], and one study investigated recurrent climate exposure [63].

#### Aquatic impacts

In the Mediterranean, a decrease in soil water content due to compound hot-dry events, especially in the winter and spring, has resulted in increased forest mortality, decreased crop yields, and increased vegetation stress [66]. Compound hot-dry events occur earlier in the year (i.e., spring and early summer), which affects the growing season length and decreases ecosystem productivity [66]. Furthermore, parts of the Mediterranean (i.e., Italy and Spain) are known for olive production, which will be impacted if warming reaches above 2 ºC [66].

#### Terrestrial impacts

Sweden and the Netherlands have experienced the loss of land due to flood-induced coastal erosion and rising sea levels [63]. Flooding from high tides occurs more frequently, causing disruption to those regions and resulting in $240 billion of assets exposed to recurrent flooding [63]. In both of these regions, compound flood events are more prevalent in densely populated areas, which are often found in low-elevation zones; higher elevations are less hospitable, meaning the communities cannot move higher to escape the changing conditions. Extreme storms and high river discharges compound the negative impacts of recurrent flooding in these communities [63].

The increasing prevalence of hot-dry compound climate events is changing species distributions, especially forest and grassland species, with mountain grasslands proving highly susceptible. For instance, European forests are changing due to both anthropogenic (e.g., fire-tolerant species) and non-anthropogenic (e.g., climatic changes) efforts [61]. In China, increasing compound wet-dry events in high population areas throughout the Yangtze River Valley have impacted the ecology of the agricultural and pastoral transition zone, arid and semi-arid areas, and the Qinghai–Tibet Plateau [62]. Other examples include fire events, triggered by an increase in compound hot and dry conditions in the Pantanal region of Xingu, Brazil, posing threats to production and communities who work or live in that area [65]. Lastly, in California, compound heatwave events are causing increased dust emissions due to vegetation decay [64].

## Discussion and future recommendations

This scoping review aims to understand the current state of the literature on CCEs and their impacts on the societal-environment interface. The results identified impacts on agriculture systems (e.g., crop yield, farmholders, livestock), infrastructure (e.g., public facilities, industries, flood management systems), public health (e.g., mental health, water quality, infectious diseases), and habitat loss. Using our strict inclusion criteria of empirical research explicitly addressing CCEs and their societal implications, only 28 studies were included, demonstrating that more studies are needed that directly addresses CCEs, despite a large number of studies focusing on single extreme climate events.

Like other reviews, such as Barquet et al. [35], we note a lack of consistent use of concepts and methods, which limits the analytical significance, the potential synthesization of research evidence, and ultimately the implementation of science into real-world settings. As more formal definitions emerge, we recommend terms from government sources and international organizations like the US 5th National Climate Assessment [13]. As CCE terms become more solidified, retrospective analysis to understand the impacts of previous CCEs, often not labeled as a CCE by authors, is needed. At the time of this review, an emerging analysis to categorize previous climate hazards as CCEs is underway and has shown that 19% of global disasters from 1900 to 2023 could be considered multi-hazard, but they are disproportionately responsible for nearly 59% of the estimated global economic losses [24].

**Fig. 4:**
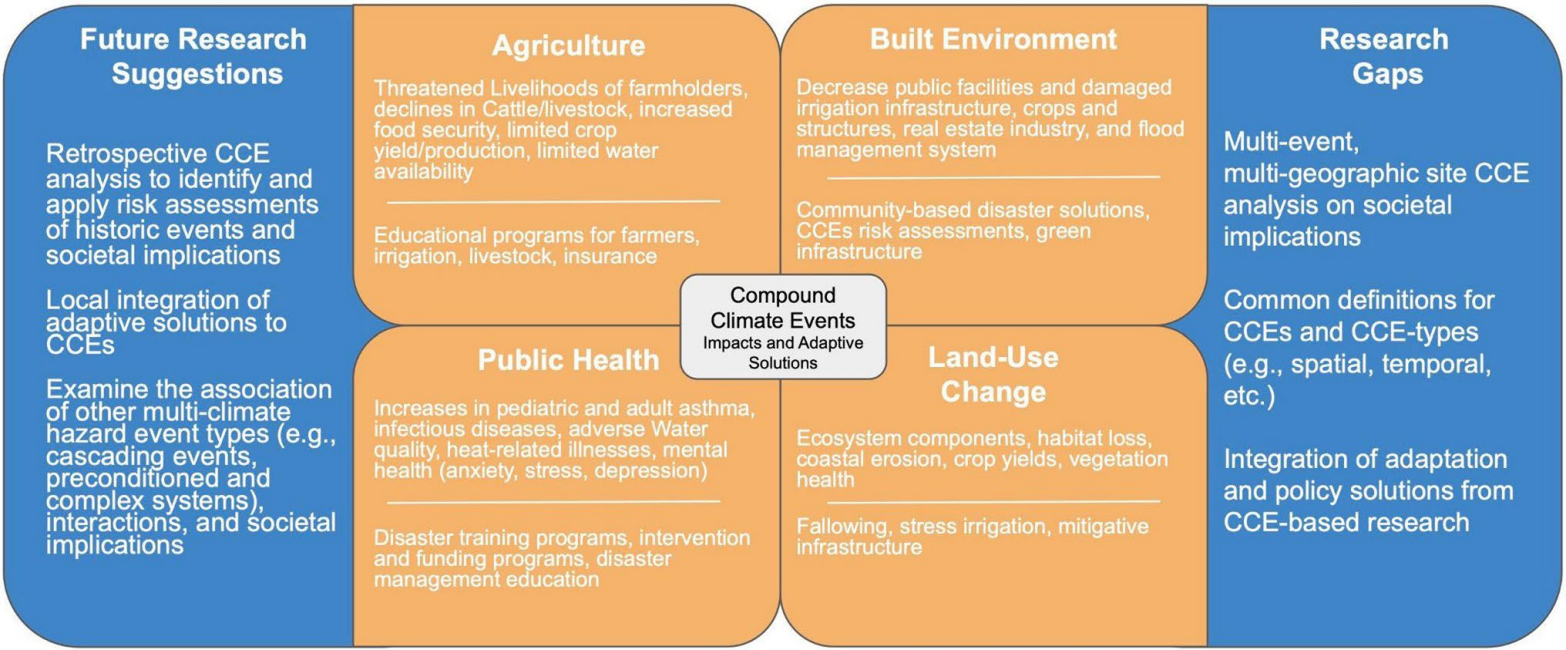
Conceptual framework for the scoping review study focusing on societal impacts of compound climate events (CCE). Provides future research suggestions and identifies CCEs impacts, adaptive solutions, and research gaps

A critical aspect of this scoping review analysis is providing future research recommendations and policies for societal impacts on CCEs. Furthering this knowledge will assist in understanding the extent to which CCEs disrupt society, thereby providing a starting place for comprehensive discussions between communities to adopt new climate policies as CCEs become more frequent and intense [14, 24, 56]. Understanding the impacts of CCEs requires interdisciplinary collaboration across different fields of study and sectors (e.g., industry, government), emphasizing the importance of a common conceptual framework. We provide this preliminary guidance in Fig. 4, representing key findings for each sector and future research needs. Table 1, which provides common cross-disciplinary definitions that can be used across sectors specific to CCEs.

In addition, although bottom-up approaches and single CCE case studies are key components of disaster research and a recommended approach for CCE risk assessment [20, 21, 24, 27, 28], methodological advances are still needed to study the societal implications across a large sample of CCEs as it can be difficult to aggregate or compare evidence across single-CCE analysis because of differences in study methodology [67, 68]. Analysis across geographic contexts and multiple years can supplement single CCE case studies by providing more precise estimates of the typical association between CCE exposure and societal impacts, increasing understanding of the heterogeneity across associations between different CCE events and locations with differing underlying resilience, and consistency of the association across CCE-types [68–71].

### Agriculture

Research in the agricultural sector highlights the need for adaptive strategies to address challenges created by CCEs (e.g. food insecurity, livestock loss, crop yield decline). Adaptive solutions should be applied at all levels, from seed development (e.g., genetic traits for survival to CCEs) to regional programs educating farmers [48, 71]. Policies must reduce CCE-related risks and uncertainty while supporting time and cost-dependent techniques like crop choice, irrigation, livestock shifts, and insurance [46, 72–74]. Disseminating these strategies is crucial to prevent harmful coping mechanisms, such as excessive fertilizer use or off-farm employment, which can further harm agriculture [41, 44–46, 48, 72, 74, 75].

Research is further needed to implement assessment methodologies like Guo et al. [76], that identify CCEs occurrence and can assess impacts to agriculture (e.g. crop damage). Additionally, with the exception of research by Zhao et al. [71] and Budhathoki et al. [72], most CCE work has focused on recurrent droughts, highlighting the need to examine other CCE-types in detail (e.g., Flood-Heat, Drought-Flood, Frost-Drought, etc.) and the risk management and policy measures (e.g., crop choice, irrigation) required for these types of events.

### Public health

At the time of this scoping review, research examining CCEs found these events as risk multipliers, with increases in damage to infrastructure and decreases in healthcare access [50, 54]. Echoing Leppold et al. [25], a scoping review focused on public health and CCEs, we recommend tailored intervention programs for CCEs, as these differ from single disasters and require integrated strategies for preparedness, response, and recovery across CCEs. Intervention programs, such as the Federal Emergency Management Agency, that provide continued and potential larger amounts of funds, and disaster management education to help support families' health needs (e.g., food, mosquito repellent) following CCEs [9]. Policies are also needed to improve disaster training for healthcare workers and address gender equity that is amplified by CCEs. In locations with advanced healthcare systems, healthcare accessibility during disaster events and ensuring continuity of healthcare for individuals with pre-existing conditions is a key policy and research need [50, 54]. Overall, more research is needed to identify health conditions with respect to assessing CCEs and CCE-event types (i.e., heatwave, tropical cyclone, wildfire), using quantitative analysis, in order to better understand which populations should be priority for intervention, as the majority of studies to date have been conducted using surveys [51, 53], questionnaires [50, 55] and structured interviews [51].

### Built environment

A recurring theme in studies concerning the built environment is the critical need for targeted infrastructure policies to mitigate the impacts of CCEs that have severely affected the built environment, particularly in areas with limited resources and inadequate support systems. The research included in this scoping review highlighted a number of opportunities, including understanding groundwater flooding [57], collaboration between communities[56], and the potential for green infrastructure in mitigating CCEs [77]. It is evident that governing bodies play a central role in promoting community engagement and enacting innovative infrastructure legislation to bolster societal structural integrity. A notable example from this scoping review was Puerto Rico, which grapples with the complex challenge of recovering from compounding hurricanes and has created participatory pathways to provide opportunities for community-based disaster solutions by improving decision-making capacity [78]. This framework which encourages co-production of knowledge can be applied to other locations globally to enhance their response to CCEs. Like our review, previous work on CCEs and infrastructure, has shown a gap between scientific approaches and practical implementation at the local level [35]. However, methodologies for local application, like Stablein et al. [78], should consider practical restraints (e.g., municipal, data availability, financial resources, etc.) [35, 79]. Methods for CCE risk assessments should also be further investigated. Such as Fernedez-Perez et al.’s [80] study that implements a novel risk analysis methodology for local port infrastructures and accounts for complex interactions between subsystems (e.g. infrastructure system, socioeconomic system) due to CCEs.

### Land use change

CCEs significantly impact land-use increasing risk for interconnections between land, cultural heritage, community well-being, and other societal sectors pertaining to agriculture and the built environment. The negative impacts of CCEs on land use include loss of land [63], soil degradation [64, 66], reductions in agriculture output [66, 81, 82], and increases in fire risk [65]. More research is needed to examine biome changes resulting from land use alterations triggered by CCEs, such as mapping changes in semi-arid and arid lands to identify at-risk areas and implement preparatory measures [62]. Mitigative strategies and policies are needed to help combat CCEs risks rather than single CCE risks for water resources and urbanizing areas due to land-use change [49, 83].

### Limitations

The papers included in this scoping review were determined by the search criteria and terms we used, as shown in Supplemental Table 1. For this reason, there could be other studies that examined CCEs, but were not included in this review. We recommend future work, such as a systematic review that captures CCEs in more detail than a scoping review, which is tasked as a method to “scope” out key knowledge gaps, clarify key concepts and definitions, as well as, inform future research, policy, and practice through a limited number of publications [84–86]. This method is appropriate given the emerging nature of CCEs and how CCE impacts have been studied by diverse disciplines and methods. Second, our search strategy focused on articles using the term "compound" and other umbrella terms (e.g., recurrent, multi-hazard), that may have missed other important terminology, limiting our ability to include all potential combinations of climate hazard types. Consequently, this review identified only cases explicitly described as CCEs, although additional studies likely address multi-disaster climate events without explicitly labeling them as such. This limitation is also found in other multi-disaster reviews, such as Leppold et al. [25] and Barquet et al. [35].

Lastly, emerging research has begun to explore the implications of cascading events, a term not addressed in our study. This term is considered ambiguous, as it can refer to a variety of pathways, including chains of events leading to a hazard, interactions between events, direct impacts, and the subsequent consequences of these impacts [35]. Although this term is not part of the US National Climate Assessment [13], future work is still needed to understand the implications of these events compared to more traditional CCEs, as their societal implications may include more significant societal implications.

## Conclusion

This scoping review explores the complex relationship between compound climate events (CCEs) and their impacts on the societal-environment interface, highlighting key areas for further research and policy. Despite rising awareness of CCEs' increasing frequency and intensity [1] our analysis found limited studies focused on their societal implications. Consistent terminology and new research on CCE impacts and adaptation strategies are crucial. Additionally, efforts should be made to retroactively identify past CCEs by examining multiple CCEs, rather than single case-study analysis of CCE events, to find a more precise estimate of the association of CCE exposure and societal impacts. Overall, this review emphasizes the urgent need to deepen our understanding of CCEs to guide resilient policies and effective mitigation strategies.

## Supplementary Information

Below is the link to the electronic supplementary material.

Supplementary material 1.

Supplementary material 2.

## Data Availability

Data provided in supplemental documents.

## Abbreviations

IPCC: Intergovernmental panel on climate change
CCE: Compound climatic event
PRISMA: Preferred reporting items for systematic reviews and meta-analyses
CCWE: Compound coastal water events
