# Supplementary material for: Scoping review of the societal impacts of compound climate events
Source: Discov Environ. 2025 Jan 16;3(1):2. doi: 10.1007/s44274-025-00185-y (PMC11739198; doi:10.1007/s44274-025-00185-y)
Supplement: Supplementary file 2 — Supplementary material 2. [file 44274_2025_185_MOESM2_ESM.docx]

**Supplemental Table 1.** A summary of the search terms that were used in this scoping review for each of the categories that are based on the inclusion criteria. These search terms were used in the databases with the ‘AND’ operation. For instance, “compound*” AND “climate” AND “well-being” as one search string.

| **Search Terms Syntax** | |
| --- | --- |
| **Agriculture** | “Climate” AND “compound” AND “food supply”; “Climate” AND “compound” AND “agriculture”; “Climate” AND “compound” AND “food industry”; “Climate” AND “compound” AND “crop yield”; “Climate” AND “compound” AND “farm workers”; “Climate” AND “compound” AND “farm labor”; “Climate” AND “compound” AND “farm practices”; “Climate” AND “compound” AND “cash crops”; “Climate” AND “compound” AND “family farms”; “Climate” AND “compound” AND “food security”; “Climate” AND “compound” AND “food”; “Climate” AND “compound” AND “crop production”; “Climate” AND “compound” AND “crop cultivation”; “Climate” AND “compound” AND “food safety”; “Climate” AND “compound” AND “agriculture damage”; “Climate” AND “compound” AND “agriculture workers”; “Climate” AND “recurrent” AND “food supply”; “Climate” AND “recurrent” AND “agriculture”; “Climate” AND “recurrent” AND “food industry”; “Climate” AND “recurrent” AND “crop yield”; “Climate” AND “recurrent” AND “farm workers”; “Climate” AND “recurrent” AND “farm labor”; “Climate” AND “recurrent” AND “farm practices”; “Climate” AND “recurrent” AND “cash crops”; “Climate” AND “recurrent” AND “family farms”; “Climate” AND “recurrent” AND “food security”; “Climate” AND “recurrent” AND “food”; “Climate” AND “recurrent” AND “crop production”; “Climate” AND “recurrent” AND “crop cultivation”; “Climate” AND “recurrent” AND “food safety”; “Climate” AND “recurrent” AND “agriculture damage”; “Climate” AND “recurrent” AND “agriculture workers”; “Climate” AND “multi-hazard” AND “food supply”; “Climate” AND “multi-hazard” AND “agriculture”; “Climate” AND “multi-hazard” AND “food industry”; “Climate” AND “multi-hazard” AND “crop yield”; “Climate” AND “multi-hazard” AND “farm workers”; “Climate” AND “multi-hazard” AND “farm labor”; “Climate” AND “multi-hazard” AND “farm practices”; “Climate” AND “multi-hazard” AND “cash crops”; “Climate” AND “multi-hazard” AND “family farms”; “Climate” AND “multi-hazard” AND “food security”; “Climate” AND “multi-hazard” AND “food”; “Climate” AND “multi-hazard” AND “crop production”; “Climate” AND “multi-hazard” AND “crop cultivation”; “Climate” AND “multi-hazard” AND “food safety”; “Climate” AND “multi-hazard” AND “agriculture damage”; “Climate” AND “multi-hazard” AND “agriculture workers” |
| **Built Environment** | “Compound Disaster” AND “infrastructure”; “Recurrent Disaster” AND “infrastructure”; “Multiple Disaster” AND “infrastructure”; “Cascading Disaster” AND “infrastructure”; “Overlapping Disaster” AND “infrastructure”; “Multi-hazard Disaster” AND “infrastructure”; “Compound Disaster” AND “water systems”; “Recurrent Disaster” AND “water systems”; “Multiple Disaster” AND “water systems”; “Cascading Disaster” AND “water systems”; “Overlapping Disaster” AND “water systems”; “Multi-hazard Disaster” AND “water systems”; “Compound Disaster” AND “transportation system”; “Recurrent Disaster” AND “transportation system”; “Multiple Disaster” AND “transportation system”; “Cascading Disaster” AND “transportation system”; “Overlapping Disaster” AND “transportation system”; “Multi-hazard Disaster” AND “transportation system”; “Compound Disaster” AND “electricity”; “Recurrent Disaster” AND “electricity”; “Multiple Disaster” AND “electricity”; “Cascading Disaster” AND “electricity”; “Overlapping Disaster” AND “electricity”; “Multi-hazard Disaster” AND “electricity”; “Climate” AND “Compound” AND “infrastructure”; “Climate” AND “Recurrent” AND “infrastructure”; “Climate” AND “Multi-hazard” AND “infrastructure”; “Climate” AND “Compound” AND “water systems”; “Climate” AND “Recurrent” AND “water systems”; “Climate” AND “Multi-hazard” AND “water systems”; “Climate” AND “Compound” AND “transportation system”; “Climate” AND “Recurrent” AND “transportation system”; “Climate” AND “Multi-hazard” AND “transportation system”; “Climate” AND “Compound” AND “electricity”; “Climate” AND “Recurrent” AND “electricity”; “Climate” AND “Multi-hazard” AND “electricity” |
| **Public Health** | “Compound” AND “Mental Health”; “Compound Disaster” AND “Mental Health”; “Compound Disaster” AND “Depression”; “Compound Disaster” AND “Anxiety”; “Cascading Disaster” AND “Mental Health”; “Multi-Hazard” AND “Mental Health”; “Climate” AND “Compound” AND “Mental Health”; “Climate” AND “Recurrent” AND “Mental Health”; “Climate” AND “Recurrent” AND “Depression”; “Climate” AND “Recurrent” AND “Anxiety”; “Compound Disaster” AND “PTSD”; “Cascading Disaster” AND “Mental Health”; “Recurrent Disaster” AND “Mental Health”; “Climate” AND “Multi-hazard” AND “Mental Health”; “Climate” AND “Compound” AND “Mental Health”; “Climate” AND “Compound” AND “Depression”; “Climate” AND “Compound” AND “Anxiety”; “Climate” AND “Compound” AND “PTSD”; “Climate” AND “Compound” AND “Suicide”; “Recurrent Disaster” AND “Depression”; “Recurrent Disaster” AND “Anxiety”; “Recurrent Disaster” AND “Mental Health”; “Recurrent Disaster” AND “PTSD”; “Multiple Disaster” AND “Mental Health”; “Multiple Disaster” AND “Depression”; “Multiple Disaster” AND “Anxiety”; “Multiple Disaster” AND “PTSD”; “Multiple Disaster” AND “Suicide”; “Multi-Hazard Disaster” AND “Mental Health”; “Compound Disaster” AND “physical health”; “Recurrent Disaster” AND “physical health”; “Multiple Disaster” AND “physical health”; “Cascading Disaster” AND “physical health”; “Overlapping Disaster” AND “physical health”; “Multi-Hazard Disaster” AND “physical health”; “Compound Disaster” AND “exercise”; “Recurrent Disaster” AND “exercise”; “Multiple Disaster” AND “exercise”; “Cascading Disaster” AND “exercise”; “Overlapping Disaster” AND “exercise”; “Multi-Hazard Disaster” AND “exercise”; “Compound Disaster” AND “public health”; “Recurrent Disaster” AND “public health”; “Multiple Disaster” AND “public health”; “Cascading Disaster” AND “public health”; “Overlapping Disaster” AND “public health”; “Multi-Hazard Disaster” AND “public health”; “Climate” AND “Compound” AND “community greenspaces”; “Climate” AND “Recurrent” AND “exercise; “Climate” AND “Recurrent” AND “physical health”; “Climate” AND “Compound” AND “physical health”; “Climate” AND “Multi-Hazard” AND “physical health; “Climate” AND “Compound” AND “public health”; “Climate” AND “Recurrent” AND “public health”; “Climate” AND “Multi-hazard” AND “public health” |
| **Land-Use Change** | “Climate” AND “Compound event” AND “Land Use Change”; “Climate” AND “Compound event” AND “Water Management”; “Climate” AND “Compound event” AND “Forest Management”; “Climate” AND “Compound event” AND “urban development”; “Climate” AND “Compound event” AND “abandoned housing”; “Climate” AND “Compound event” AND “urban development”; “Climate” AND “Recurrent” AND “Land Use Change”; “Climate” AND “Recurrent” AND “Water Management”; “Climate” AND “Recurrent” AND “Forest Management”; “Climate” AND “Recurrent” AND “urban development”; “Climate” AND “Recurrent” AND “abandoned housing”; “Climate” AND “Recurrent” AND “urban development”; “Climate” AND “multi-hazard event” AND “Land Use Change”; “Climate” AND “multi-hazard event” AND “Water Management”; “Climate” AND “multi-hazard event” AND “Forest Management”; “Climate” AND “multi-hazard event” AND “urban development”; “Climate” AND “multi-hazard event” AND “abandoned housing”; “Climate” AND “multi-hazard event” AND “urban development” |
